# Control‐value appraisals and the emergence of students' boredom: An in situ perspective within lessons

**DOI:** 10.1111/bjep.70048

**Published:** 2025-11-22

**Authors:** Richard Göllner, Kristina Kögler

**Affiliations:** ^1^ University of Potsdam Germany; ^2^ University of Stuttgart Germany

**Keywords:** appraisal‐boredom relations, control‐value theory, experience sampling

## Abstract

**Background:**

Despite a growing body of research demonstrating that control and value appraisals predict students' experiences of boredom, less attention has been paid to appraisals arising from specific learning situations and their consequences for students' emotional responses.

**Aims:**

In the present study, we disentangled students' individual differences from their momentary learning experiences to examine appraisal–boredom relations, including their reciprocal effects.

**Methods:**

We analysed experience‐sampling data from *N* = 95 secondary school students who provided repeated ratings of their comprehension, interest, and boredom during eight lessons over two consecutive curricular weeks. The data were examined using multilevel structural equation modelling with cross‐lagged relations for students' momentary experiences.

**Results and Conclusions:**

We found that higher interest was consistently associated with lower boredom. In contrast, comprehension showed a more complex pattern. Boredom due to overchallenge appeared to stem from stable individual differences, whereas boredom resulting from underchallenge emerged from students' momentary comprehension. Finally, analyses of reciprocal relations revealed that boredom experienced toward the end of a lesson predicted decreases in students' subsequent comprehension and interest, highlighting the potential for downwards spirals of disengagement within the classroom context.

**Key points**

- Students' interest was consistently associated with lower levels of boredom across all levels of analysis.
- Students' perceived comprehensibility showed a non‐linear relationship with boredom, reflecting both overchallenge and underchallenge as sources of boredom.
- Reciprocal relations revealed that boredom predicted lower subsequent comprehension and interest, though these effects were not consistent throughout the lesson.

## INTRODUCTION

Students' boredom is one of the most prevalent emotions and is typically described as an unpleasant and undesired feeling accompanied by low psychological arousal and a lack of stimulation (Pekrun, 2006; Pekrun et al., 2014). In school, students are bored up to 50% of the time during lessons, which in turn has been shown to be associated with attention problems, lower intrinsic motivation, and overall negative consequences for students' learning achievement (Goetz et al., 2007; Nett et al., 2011; Pekrun, 2006; Pekrun et al., 2014, 2017; Putwain et al., 2022).

According to appraisal theories like the control‐value theory (Pekrun, 2006), boredom results when students perceive low subjective control over a task (control appraisal) and do not value the task that they are working on (value appraisal). Though the relevance of control and value appraisals for explaining learners' boredom is generally accepted, the empirical evidence is contradictory to some extent. Whereas value appraisals consistently show a negative relationship with boredom, control appraisals do not appear to be strongly linked to reduced boredom (Bieg et al., 2013; Golle et al., 2022; Kögler & Göllner, 2018; Pekrun et al., 2010; Raccanello et al., 2022). According to the control‐value theory of achievement emotions, boredom can result from both low control (i.e., overchallenge) and high control (i.e., underchallenge, Pekrun, 2006). Similarly, Csikszentmihalyi (2000) suggests that an optimal balance between challenge and skill is crucial for engagement, with both excessive challenge and insufficient challenge potentially leading to disengagement. This dual effect may explain why control appraisals show less consistent relationships with boredom compared to value appraisals.

At the same time, previous research mainly addressed students' appraisals at the person level without taking into account the specific learning situation in which appraisals are expected to arise. This makes it more difficult to understand how students' boredom emerges from their specific learning experiences and how different appraisals contribute to its development. For this reason, it seems a worthwhile endeavour to further elucidate the relation between control and value appraisals and their effects on the emergence of boredom in situ. In addition, most studies have examined appraisals solely as predictors of students' academic emotions, neglecting the potential reciprocal associations between boredom and control‐value appraisals. This approach fails to capture the dynamic nature of emotions (see also Goetz et al., 2020). It is highly plausible that appraisals do not only predict boredom, but that boredom can also directly influence learners' subsequent learning experiences and their appraisals. This is particularly relevant for boredom, an emotion inherently associated with discomfort and a tendency to seek alternative activities (Pekrun et al., 2010). As such, boredom has been shown to form reciprocal relationships with learners' subsequent appraisals (Forsblom et al., 2022; Pekrun et al., 2014).

In the present study, we used an intense momentary assessment of students' perceived comprehension and interest and their boredom during school lessons. Applying an experience sampling approach allowed us to examine the relationship between appraisals in a specific learning situation and their potential consequences for students' boredom while controlling for students' interindividual differences. In addition, we addressed the reciprocal associations between students' perceived comprehension, interest and their reported boredom during their actual learning and also took care to consider non‐linearity in the appraisal–boredom relationship.

### Control and value appraisals as predictors of students' boredom

Considering the significance of emotions in learners' academic achievement (Camacho‐Morles et al., 2019; Pekrun, 2017; Tze et al., 2016), it is crucial to comprehend the emergence of these emotional experiences in learning situations. In this context, appraisal theories of emotion (Arnold, 1960; Ellsworth, 2013) have demonstrated considerable influence. The basic idea of appraisal theories is that emotions emerge and develop due to appraisals of the perceived goal relevance, goal in/congruence, un/expectedness, control and agency (Moors, 2017). The control‐value theory as a core theoretical framework (Pekrun, 2006) posits two main individual determinants of academic emotions, including boredom. Specifically, learners who feel capable of successfully performing a task without perceiving it as too easy or too difficult and who subjectively value the task for intrinsic or extrinsic reasons will be less likely to feel bored. Previous research has revealed that control and value appraisals are associated with achievement emotions. For instance, findings from cross‐sectional studies have shown that academic self‐concept, which gives rise to strong perceived control, is positively correlated with enjoyment and negatively correlated with boredom at university (Pekrun et al., 2010) as well as secondary school (Bieg et al., 2013; Goetz et al., 2006). In the same vein, Pekrun et al. (2010) found negative relations between both control and value appraisals and academic boredom at university. These relations have also been confirmed in research using longitudinal study designs. For instance, a study conducted by Putwain et al. (2018) among primary school students with three waves separated by 3‐month intervals showed that control and value in terms of students' self‐concept and interest in mathematics negatively predicted boredom at the next measurement point.

### The situation specificity of students' boredom

According to appraisal theories, control and value appraisals as well as students' achievement emotions originate from an individual's evaluation of a specific situation. Appraisal theory assumes emotions to depend on how a concrete situation is interpreted along several appraisal dimensions, including the importance of the situation, expectations that one will be able to cope with it, and the extent to which it is possible to control the outcome (Moors, 2017; Pekrun et al., 2010; Roseman & Smith, 2001).

Learning situations have mainly been framed in terms of achievement domain specificity. Specifically, previous research has shown that students' achievement emotions such as anxiety, enjoyment or boredom are only weakly correlated across different domains, such as mathematics and language arts. This has led to the conclusion that students' experiences in a given domain give rise to domain‐specific emotional responses (Goetz et al., 2006). This principle of specificity might be extended to concrete learning situations *within* an achievement domain. For example, Tanaka and Murayama (2014) conducted an ambulatory assessment involving 12 repeated self‐report measurements from a sample of 158 undergraduate students to examine the within‐person relationships between task‐specific perceptions (i.e., expectancy, utility and difficulty) and the emotions of interest and boredom. The assessments took place after each class in a 12‐week university psychology course. They found that a higher perception of both expectancy and utility, as well as a lower perception of difficulty, was associated with higher interest and lower boredom levels within students. Though these findings supported the relevance of situational experiences for students' achievement emotions, several aspects remain unresolved.

First, previous research has primarily focused on value appraisals, particularly in terms of interest (Loukomies et al., 2015), while paying less attention to control appraisals in specific learning situations. Control appraisals are typically conceptualized as individuals' beliefs in their general ability to manage tasks and challenges (Frenzel et al., 2007), which makes it more difficult to capture the specificity of a given situation. Moreover, conventional operationalizations of control appraisals such as self‐concept or self‐efficacy may be less suited to capturing moment‐to‐moment fluctuations during shorter timeframes, such as during a lesson. This is particularly true for self‐concept, which is generally less context‐specific compared to self‐efficacy (Bong & Skaalvik, 2003; Ferla et al., 2009). In line with this, the study by Tanaka and Murayama (2014) argued that individuals' task perceptions about how easy it will be to understand the course material are similar to perceptions of control, with greater emphasis on the task than the self.

Second, existing research using experience sampling does often not allow for testing the relationships between appraisals and boredom *within* one learning unit. For instance, research designs often rely on repeated measures across lessons or days, which makes it difficult to capture the situational dynamics between appraisals and boredom, as the context in which learning occurs continuously varies. In fact, any prediction of students' boredom in one lesson by their appraisals from the lesson or day before reveals more about students' individual tendency to appraise learning situations in general instead of their specific learning experiences (Ahmed et al., 2010; Nett et al., 2017; Tanaka & Murayama, 2014).

Third, the characteristics of control and value appraisals themselves are less frequently considered than one might expect. That is, control and value appraisals are seen as equally contributing to the emergence of student achievement emotions in general and the emergence of boredom specifically. On the other hand, existing findings tend to show a weaker and less consistent relationship between control appraisals and boredom compared to value appraisals (Raccanello et al., 2022). According to control‐value theory, one possible explanation for this could be the presence of non‐linear relationships between appraisals and students' boredom (see Pekrun & Loderer, 2020; Shao et al., 2020). Perceiving a task as too easy may be just as strongly linked to boredom as perceiving it as too difficult (Acee et al., 2010; Krannich et al., 2019; van Tilburg & Igou, 2012; Westgate & Wilson, 2018).

### Reciprocal appraisal‐emotion relations

Previous research on the relationship between appraisals and boredom was mainly guided by the idea that appraisals drive the emotion process. However, it is reasonable to assume a more dynamic interplay between appraisals and achievement emotions, particularly in the case of boredom, as it is fundamentally different from other motivational or emotional constructs such as interest, engagement or enjoyment (Pekrun, 2006; Pekrun et al., 2014). This becomes most apparent when regarding the distinction between interest and boredom. Whereas a lack of interest reflects a neutral emotional state, which is not necessarily experienced as aversive or ‘painful’ (Pekrun et al., 2010; Vodanovich, 2003), boredom has been characterized as a negative feeling and comes along with a salient aversion to continuing to feel bored. Experiencing boredom is related to unwillingness to remain in a situation and is assumed to trigger the impulse to leave it (Daschmann et al., 2011). Accordingly, experiencing boredom is likely to influence how subsequent learning experiences are evaluated. For instance, students who become bored because they perceive a learning situation as too difficult or insufficiently interesting might shift their attention away from the learning content, which in turn hampers their control and interest in the further course of the same lesson. Consequently, appraisals and boredom may create a mutually reinforcing cycle of reciprocal associations. A study conducted with secondary school students confirmed these reciprocal relations between three consecutive annual assessments, demonstrating that lower control appraisals in terms of students' competence beliefs in mathematics were associated with higher levels of boredom, which in turn, were related to lower competence beliefs (Forsblom et al., 2022).

In sum, a critical test of the relationship between students' control and value appraisals and the emergence of students' boredom needs to be conducted in situations where these appraisals occur and boredom arises. In the present study, we focused on students' learning experiences during lessons to address the situational nature of boredom. In addition, assuming that boredom is solely the consequence of appraisals may overlook the effect of boredom on how learners perceive upcoming learning situations. Therefore, in our analysis we also considered the reciprocal relationships between control, value and students' boredom.

### The present study

In the present study, we used experience sampling data to follow students over the course of eight lessons, during which they were asked about their learning experiences at multiple time points within each lesson. This design allowed us to isolate specific moments of learning by accounting for situationally unspecific differences, such as those attributable to individual differences between students or variations across lessons. Thereby, the within‐lesson relationships are interpreted relative to already controlled interindividual differences between students. The analytic view on variations across lessons also made it possible to examine the relationship between appraisals and boredom at the level of students' more general learning experiences. We formulated the following hypotheses.

First, we hypothesized that students' reported comprehension and their interest would be negatively related to their boredom at the student level and the lesson level. Students who generally experience lower comprehensibility and less interest across specific learning situations were expected to report higher levels of boredom (Hypothesis 1a). Given the nature of comprehension and interest, we further assumed that comprehensibility would be less closely related to boredom than students' interest (Hypothesis 1b). Additionally, we explored the possibility of a non‐linear relationship between students' perceived comprehension and boredom (Hypothesis 1c).^1^

Second, we hypothesized that boredom would be negatively related to comprehension and interest within specific learning situations during lessons. Students who report lower comprehension and interest in a given learning situation are expected to experience higher levels of boredom in that same situation (Hypothesis 2a). We also hypothesized that perceived comprehension and interest in one learning situation would predict students' boredom in the subsequent learning situation (Hypothesis 2b). Furthermore, we anticipated that interest would exhibit a stronger relationship with boredom than comprehension (Hypothesis 2c) and again explored the potential non‐linear relationship between comprehension and boredom (Hypothesis 2d).

Finally, we examined reciprocal relationships over the course of a lesson. We hypothesized negative associations between boredom and students' later comprehension and interest. We expected that students experiencing boredom in one learning situation would exhibit reduced interest and comprehension in the following learning situation (Hypothesis 3).

## METHOD

The data we used to address our research questions stem from a study conducted at a commercial middle school in southern Germany in 2010 (see also Kärner & Kögler, 2016; Kögler, 2015; Kögler & Göllner, 2018). Commercial middle schools are pre‐vocational schools that provide general education and basic vocational training in the field of business and administration. The research presented here was not preregistered.^2^

### Sample

*N* = 95 (39 male) ninth graders from four classes (mean age *M* = 14.91 years, *SD* = 0.85) took part in the study, which took place over 2 weeks of accounting lessons in the field of business education. Within this period, the lesson content was the same in each class and concerned the basic principles of wage accounting. All participants provided written informed consent to participate in the study. Teachers were instructed to structure their lessons as usual, without making any changes. To assess students' appraisals during learning, an experience sampling approach with six equidistant measurement points within each lesson was applied (Sembill et al., 2008). The assessment covered eight school lessons over two consecutive weeks leading to 4,560 observations (95 × 6 × 8). Due to organizational constraints, data collection during the final lesson in one class could not be completed. As a result, complete data were available for *N* = 74 students, yielding a total of 4,434 observations.

### Measures

#### Experience‐sampling measures

Every student was equipped with a mobile device and had to complete short items concerning their subjective experiences on a continuous scale from 0 to 100. The measures under consideration were assessed in the same order in each of the measurement time points and the time to complete the short questionnaire amounted to a maximum of 10–20 s, so that the teaching process was hardly interrupted. Students' boredom was measured with the statement ‘I feel bored.’ (*M* = 38.21; *SD* = 32.09). Following the argumentation of Harackiewicz and Hulleman (2010), we measured situational interest as a proxy for intrinsic value and understood it as a rather motivational force (‘I'm interested in the subject matter.’; *M* = 63.25; *SD* = 18.84). Control, on the other hand, was operationalized as comprehensibility during the lesson, which refers to a rather cognitive quality (‘I understand the subject matter right now.’; *M* = 74.92; *SD* = 26.90).

#### Covariates

Interest in economic problems was assessed using 11 items (e.g., ‘For me it is of great importance to learn to better understand economic interrelationships.’; Siegfried & Wuttke, 2019; Wild & Winteler, 1990). Students rated each item on a 4‐point scale ranging from 1 (does not apply) to 4 (does apply). The scale demonstrated good internal consistency (Cronbach's *α* = .85) and was administered prior to the experience sampling period during the learning unit. In addition, students' age and gender were considered as covariates to obtain unconfounded estimates of the relationships between appraisals and boredom.

### Analytical procedure

#### Differences among students and lessons

Given the overall aim of the study, particular care was taken to ensure that the measurement time points captured students' in‐the‐moment experiences. To avoid potential confounding, we controlled for individual differences among students as well as lesson‐specific characteristics.

To this end, we employed a multilevel modelling framework to account for the hierarchical structure of the data, with repeated lessons nested within students and measurement time points nested within each lesson. The dataset was structured in long format to represent the nesting of lessons within students, and in wide format for each lesson to include the six measurement time points.

**FIGURE 1:**
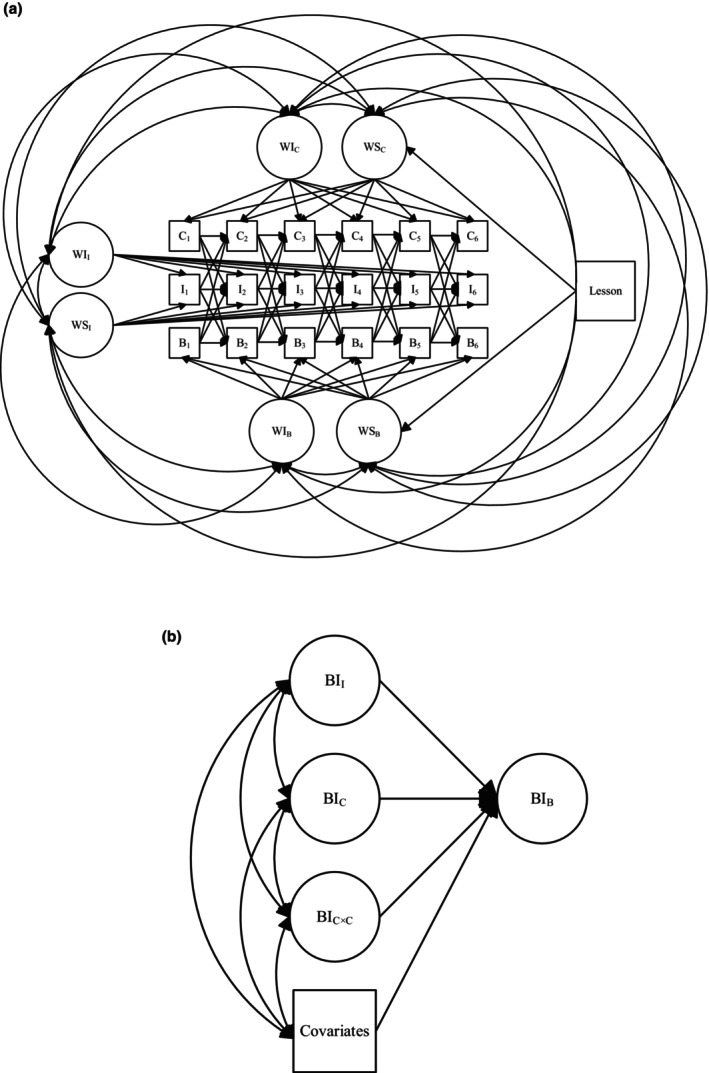
(a) Within‐student cross‐lagged‐panel model accounting for between‐lesson differences and within‐l0 lesson changes in comprehension (C), interest (I) and boredom (B). Within‐student variables (W) were modelled to capture both between‐lesson differences in mean levels (i.e., intercepts, I) and intra‐individual changes over the course of the lessons (i.e., slopes, S). Autoregressive and cross‐lagged regression paths were estimated across the six measurement points within each lesson. Between‐lesson differences were controlled using dummy‐coded variables (i.e., lesson), with the first lesson serving as the reference category. Bivariate associations between variables at each measurement time point within lessons were estimated but not additionally shown in the figure. (b) Between‐student regression model to predict students' boredom (B) by interest (I) and comprehension (C). Latent aggregates of students' lesson averages reflected between‐student differences. The squared value of students' reported comprehension (C × C) represented the aggregate of the squared comprehension scores computed at each measurement time point. Covariates included students' interest in economic problems, age and gender.

Given this, the multilevel nature of the data was addressed as follows. First, the nesting of measurement time points within lessons was modelled using latent growth curve modelling (Willett & Sayer, 1994), with intercept factors capturing overall levels and slope factors capturing the temporal trajectories of students' appraisals and boredom (see Figure 1a). The slope factor loadings were specified at equidistant intervals (−2.5, −1.5, −.5, .5, 1.5, 2.5), so that the intercept factor reflected average appraisals and boredom across one lesson, whereas the slope factor represented systematic change over the course of the lesson. To examine potential non‐linear patterns of change, we additionally tested alternative model specifications in which the loadings for the second through fifth time points were freely estimated. Second, the nesting of lessons within students was taken into account by regressing both the intercept and slope factors on a set of dummy‐coded lesson indicators, using the first lesson as the reference category. This allowed us to capture between‐lesson differences on both average levels of appraisals and boredom and their within‐lesson trajectories, while nesting these differences within individual students. These within‐level associations are interpreted relative to each student's average levels of appraisals and boredom. Finally, the nesting of lessons and measurement time points within students was modelled as the within part of an ordinary two‐level model, whereas students' individual differences were modelled at the between level. Specifically, the intercept factors were aggregated to the student level using the doubly latent approach proposed by Marsh et al. (2009). At the student level, we tested whether students' average comprehension and interest predicted their average boredom (see Figure 1b).

#### Modelling the relations between appraisals and boredom

Due to the overall modelling procedure, the relationships between students' appraisals and their boredom must be considered at three levels: between‐students, between‐lessons, and within‐lessons. At the between‐students and between‐lessons levels, we examined associations between the intercepts to determine whether students' average levels of boredom could be predicted by their average appraisals.

To test for a potential non‐linear relationship between comprehension and boredom, we computed an additional variable at each of the six measurement time points, representing the squared value of students' reported comprehension. This approach follows the standard product‐term method for modelling non‐linearity in regression analysis (see Jackman et al., 2011, for an overview). These squared terms were included in the same modelling framework as the original variables, while controlling for both interindividual differences among students and variation across lessons.

At the within‐lesson level, we applied a cross‐lagged modelling approach to examine both prospective and reciprocal relations between students' appraisals and boredom, while accounting for stable student and lesson‐level differences (Berry & Willoughby, 2017; Hamaker et al., 2015). As illustrated in Figure 1a, autoregressive paths within constructs captured the stability of students' perceptions across measurement points within a lesson, whereas cross‐lagged paths tested whether one construct at a given time point prospectively predicted another construct at the next time point.

Given the inclusion of multiple constructs and repeated measurements, as well as growth factors capturing interindividual variability, the resulting model included a substantial number of parameters. To enhance model parsimony and statistical power, we applied equality constraints to parameters across time, provided that such constraints did not lead to a significant decline in model fit.

### Missing data

To make use of all available data, we employed the robust maximum likelihood estimator in conjunction with full information maximum likelihood (FIML) procedures to handle missing data due to attrition of *N* = 21 students during the final lesson. Prior to this, we tested whether students with missing data differed from those with complete data on study variables, including covariates. The results showed no significant differences between the two groups in terms of gender distribution, *χ* ^2^(1) = .49, *p* = .480, age, *F*(1, 93) = 3.12, *p* = .080, or general interest in economic problems, *F*(1, 93) = 3.41, *p* = .070. FIML has been shown to handle missing data more effectively than traditional methods (e.g., listwise or pairwise deletion) and performs comparably to multiple imputation techniques in structural equation modelling (Jia & Wu, 2023).

The data were analysed using the statistical software M*plus* (Muthén & Muthén, 1998–2017) with the robust maximum likelihood (MLR) estimator. To determine whether the proposed model fits the data, we evaluated the model fit using the following cutoff criteria: a comparative fit index (CFI) of .95, a Tucker–Lewis index (TLI) of .95, a root mean squared error of approximation (RMSEA) lower than .08 and a standardized root mean square residual (SRMR) lower than .07 (Hu & Bentler, 1999). Model comparison was assessed using the Satorra–Bentler scaled chi‐square difference test (*TRd*).

All associations are reported in their standardized form. Statistical tests were conducted two‐tailed and with an *α*‐level of .05.

## RESULTS

### Preliminary analysis

**FIGURE 2:**
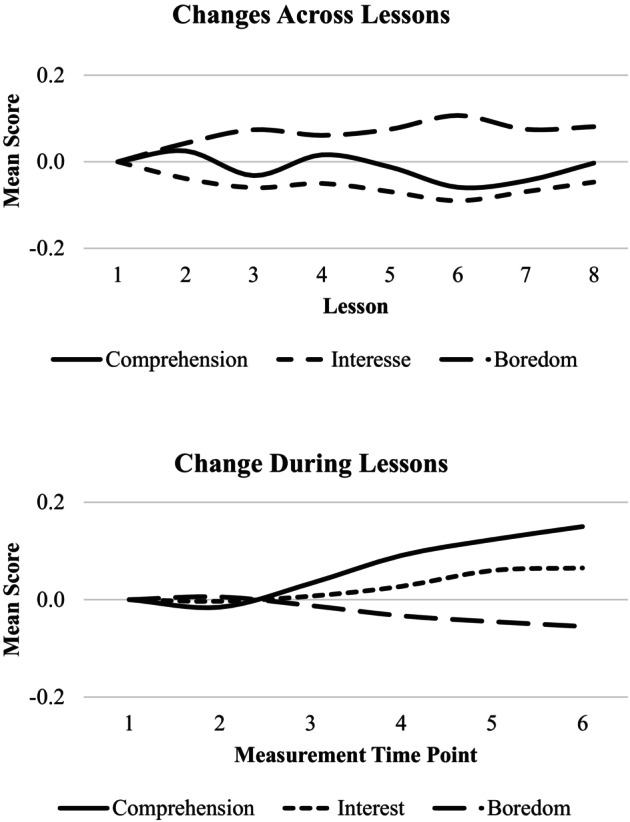
Predicted mean‐level changes across and during lessons.

Table 1 shows bivariate associations between variables across measurement time points. Before addressing our hypothesis, we first established latent growth models for addressing changes in students' appraisals and their boredom during lessons. We began by testing whether the assumption of linearity in students' changes over the course of a lesson was supported by the data. To do so, we compared a model including all constructs with freely estimated slope factor loadings at the within‐student level to models in which the slope loadings for the respective construct were fixed. The results showed that freeing the factor loadings did not result in any model fit improvement (see Table 2), indicating that the assumption of linear change holds for all the variables. In addition, inspecting the time trends derived from the model with freely estimated slope factor loadings showed a nearly linear trend for each of the investigated constructs (see Figure 2). The respective slope means revealed positive time trends for comprehension and interest (*p*s < .01), while boredom declined but did not reach statistical significance (*p* = .062; see Table 3). Across the eight consecutive lessons, the trajectories of these constructs displayed more non‐linear patterns (Figure 2).

**TABLE 1:** Correlations of perceived comprehension (C), interest (I) and boredom (B) between measurement time points.

		1	2	3	4	5	6	7	8	9	10	11	12	13	14	15	16	17	18
1	C 1		.32	.22	.12	.11	.10	.43	.21	.07	.08	.12	.09	−.27	−.16	−.17	−.10	−.12	−.04
2	C 2	.87		.42	.34	.16	.18	.16	.40	.19	.15	.05	.11	−.15	−.27	−.10	−.07	−.05	−.10
3	C 3	.79	.89		.36	.20	.26	.12	.19	.40	.25	.12	.14	−.11	−.17	−.31	−.13	−.15	−.07
4	C 4	.80	.88	.91		.38	.26	.10	.18	.27	.45	.19	.23	−.07	−.16	−.21	−.33	−.14	−.14
5	C 5	.79	.87	.86	.89		.34	.15	.14	.21	.26	.40	.25	−.15	−.09	−.12	−.17	−.33	−.14
6	C 6	.75	.83	.85	.88	.88		.12	.07	.09	.13	.19	.44	−.05	−.04	−.04	−.06	−.16	−.29
7	I 1	.55	.54	.52	.53	.51	.44		.33	.28	.28	.21	.18	−.48	−.28	−.21	−.18	−.18	−.13
8	I 2	.49	.53	.49	.53	.49	.48	.90		.47	.39	.24	.22	−.25	−.49	−.31	−.25	−.21	−.16
9	I 3	.42	.51	.54	.53	.51	.51	.87	.88		.50	.27	.26	−.27	−.28	−.52	−.35	−.28	−.15
10	I 4	.44	.52	.56	.60	.56	.57	.85	.88	.92		.41	.30	−.23	−.31	−.36	−.53	−.34	−.20
11	I 5	.38	.49	.51	.53	.57	.51	.84	.86	.88	.91		.47	−.16	−.14	−.20	−.31	−.58	−.31
12	I 6	.38	.45	.49	.53	.52	.54	.83	.86	.90	.92	.90		−.12	−.19	−.15	−.25	−.44	−.62
13	B 1	−.34	−.40	−.41	−.37	−.33	−.36	−.75	−.73	−.78	−.72	−.70	−.68		.34	.29	.26	.21	.11
14	B 2	−.29	−.38	−.33	−.37	−.34	−.35	−.72	−.78	−.77	−.75	−.72	−.71	.88		.39	.35	.31	.24
15	B 3	−.31	−.38	−.38	−.42	−.34	−.39	−.72	−.77	−.81	−.79	−.72	−.76	.90	.93		.43	.31	.25
16	B 4	−.28	−.36	−.39	−.42	−.37	−.39	−.68	−.75	−.81	−.80	−.74	−.74	.86	.91	.95		.40	.37
17	B 5	−.27	−.34	−.35	−.38	−.38	−.38	−.68	−.72	−.76	−.76	−.75	−.73	.87	.90	.92	.93		.48
18	B 6	−.21	−.29	−.31	−.32	−.30	−.35	−.64	−.69	−.75	−.74	−.73	−.75	.85	.90	.93	.92	.91	
	*M*	.70	.74	.75	.76	.76	.79	.59	.62	.63	.65	.65	.66	.40	.38	.39	.38	.37	.37
	*SD*	.30	.28	.27	.25	.26	.24	.30	.30	.30	.28	.29	.28	.32	.32	.32	.32	.32	.32

*Note*: Between‐student results (*N* = 95) are presented below the diagonal; within‐student results, based on *K* = 8 lessons per student, are presented above the diagonal.

**TABLE 2:** Fit statistics for constrained and unconstrained latent growth curve models for students' appraisals and boredom at the lesson level.

	*χ* ^2^	*df*	SCF	CFI	TLI	RMSEA	SRMR_w_	SRMR_b_	*TRd*
Baseline^a^	983.52	643	.96	.971	.960	.027	.043	.060	
Comprehension	994.30	647	.96	.969	.961	.027	.043	.060	4.722, *p* = .317
Interest	989.78	647	.97	.969	.961	.027	.043	.060	2.080, *p* = .721
Boredom	984.41	647	.97	.970	.962	.026	.043	.060	.294, *p* = .990
All constrained	1003.93	659	.99	.969	.962	.027	.044	.060	8.397, *p* = .936

^a^ Baseline refers to a model with freely estimated factor loadings for the second to fifth measurement points for all slope factors. Constrained factor models were achieved by constraining the loadings of the respective slope factor to be linear (−2.5, −1.5, −.5, .5, 1.5 and 2.5) while freeing the loadings of the other slope factors. *N* = 760 observations.

**TABLE 3:** Descriptive statistics and bivariate associations between growth factors for boredom, perceived comprehension and interest at the lesson level.

		1	2	3	4	5	6
Comprehension							
1	Intercept						
2	Slope	−.24**					
Interest							
3	Intercept	.53***	−.05				
4	Slope	−.05	.56***	−.01			
Boredom							
5	Intercept	−.38***	.15	−.75***	−.04		
6	Slope	.07	−.42***	−.04	−.81***	.15	
	*M*	.02	.04***	.05*	.02**	−.06**	−.01
	*SD*	.02***	.001***	.03***	.001***	.04***	.001***

*Note*: *N* = 760 observations.

*** *p* < .001.

** *p* < .01.

* *p* < .05.

### Associations between comprehension, interest and boredom at the student and lesson level

**FIGURE 3:**
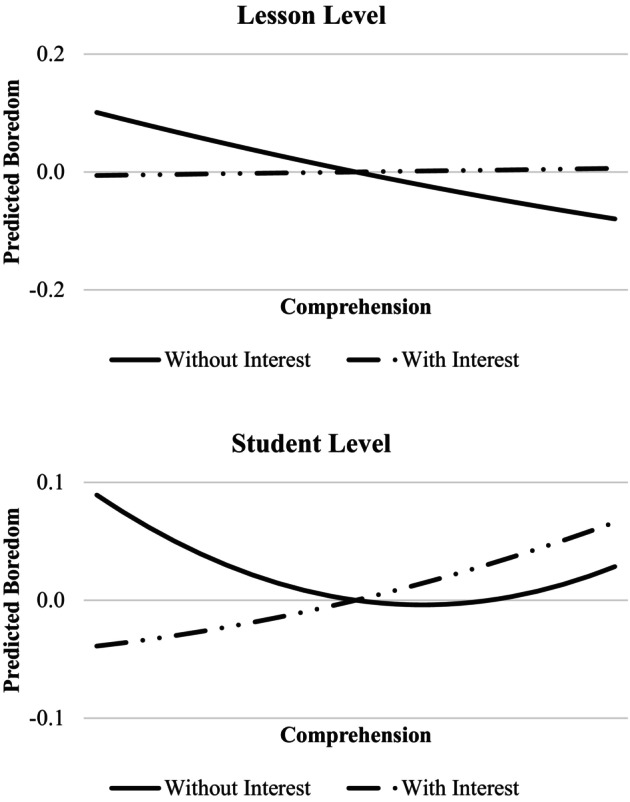
Predicted students' boredom with comprehension at the student and lesson level.

Turning to the prediction of students' boredom at both the student and lesson levels (Hypothesis 1), we examined how interest and comprehension predicted boredom at these two levels. Based on the chosen modelling strategy, we predicted intercept factors representing students' average boredom within a single lesson (lesson level), as well as the latent aggregation of these intercept factors at the student level. The results for each level are presented in Table 4. The results showed that comprehension at the student level was not linearly related to boredom but revealed a non‐linear relationship with boredom (linear: *β* = −.07, *p* = .630; quadratic: *β* = .53, *p* < .001). Students who reported either very low or very high levels of comprehension also reported higher levels of boredom (see Figure 3). Interest revealed a very strong association with boredom in a univariate model (*β* = −.83, *p* < .001). Moreover, the addition of interest reduced the previously significant non‐linear association between comprehension and boredom to a statistically non‐significant level (linear: *β* = .14, *p* = .164; quadratic: *β* = .12, *p* = .189; see Table 4).

**TABLE 4:** Predicting boredom with comprehension (C), and interest (I) at the lesson and student level.

	*β*	*SE*	*β*	*SE*	*β*	*SE*	*β*	*SE*
Lesson level (LL)								
C	−.38	.08***			−.30	.13**	.02	.07
I			−.74	.05***			−.75	.06***
C × C					.11	.10		
Student level (SL)								
C	−.44	.12***			−.07	.15	.14	.10
I			−.85	.05***			−.85	.08***
C × C					.53	.13***	.12	.09
Age	.25	.09**	.19	.08*	.20	.09*	.17	.08*
Female	−.17	.09	−.03	.06	−.23	.09**	−.04	.06
Eco. Interest	−.13	.12	.02	.05	−.03	.11	.03	.05
*R* ^2^	LL = .19 SL = .25		LL = .58 SL = .72		LL = .19 SL = .37		LL = .58 SL = .73	

*Note*: *N* = 760 observations.

*** *p* < .001.

** *p* < .01.

* *p* < .05.

To examine these associations at the lesson level, we started to inspect the interrelations among the change parameters derived from the latent growth curve model (see Table 3). Students who reported higher average levels of comprehension and interest across a given lesson also tended to report lower levels of boredom. Furthermore, more favourable changes in comprehension and interest over the course of a lesson were associated with smaller increases in students' boredom. Again, the associations between the intercepts and between the slopes for comprehension and boredom were generally weaker (intercept: *r* = −.38; slope: *r* = −.42) compared to those for interest and boredom (intercept: *r* = −.75; slope: *r* = −.81). Tests of differences between the relations with boredom for comprehension and interest indicated that both associations differed statistically significantly (*p*s < .01).

The prediction of the averaged boredom showed a linear negative relationship between comprehension and boredom (*β* = −.38, *p* < .001) and a strong negative association between interest and boredom (*β* = −.74, *p* < .001; see Table 4). When comprehension and interest were included in the same model, the linear relationship between comprehension and boredom became statistically non‐significant (*β* = .02, *p* < .782), while interest continued to show a strong negative association with boredom (*β* = −.75, *p* < .001).^3^

### Associations between comprehension, interest and boredom during lessons

Turning towards the associations within lessons at each measurement time point (Hypothesis 2), the results revealed negative associations between comprehension and boredom (−.31 ≤ *r*  ≤ −.13, *p*'s < .01) and interest and boredom (−.46 ≤ *r*  ≤ −.32, *p*s < .001) as well as positive associations between comprehension and interest (.32 ≤ *r*  ≤ .35, *p*s < .001). We then proceeded to a model which also includes autoregressive relations from one measurement point to the next, *χ* ^2^(639) = 913.70, *p* < .001; CFI = .98; TLI = .97; RMSEA = .024; SRMR_w_ = .039; SRMR_b_ = .060; scaling correction factor = .97. A model assuming equal autoregressive relations did not exhibit a lower model fit (Δ*df* = 12, *TRd* = 11.11, *p* = .520) compared with a model including freely estimated autoregressive effects. The results showed that there were statistically significant stabilities in students' boredom (*β* = .07, *p* < .05) and interest (*β* = .13, *p* < .001), whereas autoregressive effects for comprehension did not reach statistical significance (*β* = .03, *p* = .144). Next, we included cross‐time associations predicting students' boredom at one measurement point with comprehension and interest at the previous measurement point. Before, we tested whether the constraining of cross‐lagged relations led to lower model fit than a model including freely estimated cross‐lagged associations, *χ* ^2^(619) = 877.04, *p* < .001; CFI = .98; TLI = .97; RMSEA = .024; SRMR_w_ = .038; SRMR_b_ = .060; scaling correction factor = .95, which, however, was not the case (Δ*df* = 28, *TRd* = 17.52, *p* = .938). The results of the univariate models revealed a positive but statistically non‐significant association between students' comprehension and later boredom (*β* = .05, *p* = .078), while students' interest showed a statistically significant negative association with later boredom (*β* = −.08, *p* < .001). When comprehension and interest were included in the same model, comprehension continued to show a positive association with boredom (*β* = .08, *p* < .01), and interest remained negatively associated with boredom (*β* = −.12, *p* < .001). Higher interest in one learning situation was associated with a lower likelihood of experiencing boredom in the subsequent situation, whereas perceiving a learning situation as more comprehensible was associated with a higher likelihood of later boredom. Additional analyses of non‐linear effects of comprehension (*β* = −.04, *p* = .516) did not reach statistical significance. The results of a final model including constrained autoregressive as well as cross‐lagged associations are shown in Table 5.

**TABLE 5:** Predicting students' boredom (T) with previously reported boredom, perceived comprehension, interest (T−1) within lessons.

	*β*	*SE*	*β*	*SE*	*β*	*SE*	*β*	*SE*
B_(T−1)_	.09	.04*	.02	.04	.09	.04*	.04	.04
C_(T−1)_	.05	.03			.02	.05	.07	.03*
I_(T−1)_			−.09	.03**			−.11	.03***
C_(T−1)_ × C_(T−1)_					−.04	.04		
	.32 ≤ *R* ^2^ ≤ .46		.34 ≤ *R* ^2^ ≤ .48		.32 ≤ *R* ^2^ ≤ .46		.33 ≤ *R* ^2^ ≤ .48	

*Note*: *N* = 760 observations.

*** *p* < .001.

** *p* < .01.

* *p* < .05.

### Reciprocal associations between appraisals and boredom

To test the expected reciprocal relations between control‐value appraisals and boredom (Hypothesis 3), we extended the regression model by including students' boredom as a predictor of subsequent comprehension and interest. The model fit comparison showed that a model with constrained autoregressive as well as all cross‐lagged relations, *χ* ^2^(645) = 906.90, *p* < .001; CFI = .98; TLI = .97; RMSEA = .023; SRMR_w_ = .040; SRMR_b_ = .060; scaling correction factor = .98 revealed a lower model fit than a freely estimated model, *χ* ^2^(637) = 871.90, *p* < .001; CFI = .98; TLI = .97; RMSEA = .022; SRMR_w_ = .039; SRMR_b_ = .060; scaling correction factor = .97; Δ*df* = 8, *TRd* = 25.87, *p* < .01. The results of the model with freely estimated reciprocal associations yielded two main findings: First, allowing for reciprocal effects did not substantially alter the prospective relationships from comprehension (*β* = .08, *p* < .01) and interest (*β* = −.13, *p* < .001) to boredom. Second, the results revealed two negative associations from boredom to subsequent comprehension between the fourth and fifth measurement points (*β* = −.08, *p* < .01), as well as a negative association from boredom to interest between the fifth and final measurement point of the lesson (*β* = −.16, *p* < .01). All other reciprocal associations between boredom and students' appraisals were not statistically significant.

## DISCUSSION

The aim of this study was to examine the relationship between control‐value appraisals and students' boredom using a measurement approach that enabled us to focus on the specific learning situations in which boredom emerges. Our findings indicated that students who experienced higher levels of interest in the subject matter also reported lower levels of boredom overall. Students' general interest, their interest in a specific lesson, and even their momentary interest during a lesson were all negatively associated with boredom. In contrast, students' comprehension showed a non‐linear relationship with boredom. This non‐linearity appears to stem from individual differences in how students perceive the learning content as overly comprehensible, as well as from their momentary learning experiences during a lesson. The results suggest that boredom due to overchallenge is primarily linked to students' general tendency to find the content difficult to comprehend, whereas boredom resulting from underchallenge seems to arise from specific, less demanding moments during instruction. Additionally, student interest was found to buffer the negative effects of low comprehension but did not prevent boredom resulting from a lack of challenge during a lesson. Finally, the results revealed reciprocal effects of boredom on students' learning appraisals; however, these effects were less robust than initially expected.

Unlike previous research, the present study placed special emphasis on examining students' appraisals in specific learning situations and tested their associations with learners' boredom. The study design enabled us to provide new insights into how students' reported comprehension and interest relate to their experience of boredom by distinguishing between students' general tendencies to appraise learning and their momentary experiences during a lesson. The most striking findings emerged when comparing the roles of comprehension and interest. While interest consistently showed a clear negative association with boredom, the results for comprehension were more complex. Particularly important, the findings highlight the need to distinguish between students' general tendency to find a topic comprehensible and their momentary experience of comprehension during a specific lesson. At the level of students, comprehension showed a curvilinear relationship with boredom: both very low and very high levels of comprehension were associated with increased boredom. This pattern aligns with previous research (see Pekrun & Loderer, 2020; Shao et al., 2020), which suggests that learning content that is either too difficult or insufficiently challenging can lead to disengagement and the emergence of boredom (Goetz et al., 2006; Pekrun et al., 2010). In contrast, students' higher comprehension at the lesson level was consistently associated with lower boredom. That is, after accounting for individual differences, making a lesson broadly comprehensible helps prevent boredom without necessarily risking underchallenging students. However, these relationships were only present as long as students' interest was not taken into account. Once interest was included in the model, the relationship between comprehension and boredom was substantially reduced. A closer inspection of the results at the student level revealed that the inclusion of interest primarily diminished the effect of low comprehension on boredom. Students' interest appeared to compensate for the boredom associated with overly difficult learning content. This finding offers an interesting perspective on the interplay of appraisals, which are often assumed to contribute uniquely to boredom across its full range. Notably, the fact that interest can seemingly buffer the negative impact of low comprehension on boredom aligns well with well‐known theories of interest, which emphasize that interest becomes particularly relevant when there is a meaningful match between the demands of the learning context and the learner's motivational resources and states (Krapp, 2002; Silvia, 2005).

In addition, the study allowed us to examine the appraisal–boredom association at the level of specific learning experiences during a lesson. To achieve a more critical test of these associations, we investigated the extent to which appraisals made in the moment of learning predicted students' boredom as the lesson progressed. The results initially revealed that both students' interest and boredom were temporally stable within lessons. This is an intriguing finding, given that differences between students, lessons, and systematic changes occurring within a lesson had already been controlled for. The observed intra‐lesson stability suggests that students' momentary classroom experiences can trigger interest or boredom that persists throughout the lesson, highlighting the potential to influence students' achievement emotions in real time during learning (Camerman et al., 2024; Lazarides & Raufelder, 2021). Furthermore, we found that, consistent with the control‐value theory proposed by Pekrun (2006), students' appraisals arising at a given learning moment were significantly related to their experience of boredom (Camacho‐Morles et al., 2019; Pekrun et al., 2010; Raccanello et al., 2022). Students' interest during lessons emerged as a stronger predictor of boredom than their comprehension. Higher levels of interest were associated with lower boredom at the subsequent time point (Bieg et al., 2013; Pekrun et al., 2010). In contrast, students' comprehension showed a positive association with subsequent boredom. Most importantly, this finding held even when students' interest was statistically controlled for. This suggests that boredom results from a lack of challenge during specific moments of learning and thus complements results regarding comprehension at both the student and lesson levels. In this vein, the present study findings presented a new perspective on how comprehension was related to boredom and why comprehension can be expected to be less closely related to boredom than students' interest. At the same time, our results suggest that challenging learning contents or tasks during a lesson hardly result in students' boredom but should be an explicit goal for teachers in order to prevent the emergence of student boredom. Highly comprehensible learning situations seemingly risk limiting the informational gain for students, resulting in later boredom (Csikszentmihalyi, 2000; Pekrun, 2006; Westgate & Wilson, 2018).

Furthermore, our findings extend existing research by examining reciprocal relations between students' appraisals and their boredom (Putwain & Wood, 2023). According to control‐value theory, appraisals not only give rise to achievement emotions, but emotions also shape subsequent appraisals, either reinforcing or weakening them (Moors, 2013). Our results provide at least partial support for the existence of such reciprocal relations (Pekrun et al., 2017). Specifically, we found reciprocal associations between boredom and interest, as well as between boredom and comprehension, at isolated time points. Notably, boredom predicted lower interest and comprehension towards the end of lessons, raising the question of what makes this phase of instruction distinct. One possible explanation is that teachers often summarize key content and clarify expectations at the end of a lesson, prompting students to more consciously evaluate their own learning. In such evaluative moments, the experience of boredom may heighten students' awareness of their own low interest or lack of understanding.

Although the present study provided a detailed investigation of the link between students' appraisals and boredom including the modelling of non‐linear relations and the consideration of potential reciprocities, our study has limitations. Guided by appraisal theories in general and control‐value theory in specific, we employed a study design that allowed us to explore the emergence of boredom during a lesson in greater depth. However, the temporal order and causal relationships between variables cannot be conclusively determined within the scope of this study. Furthermore, all the presented findings suggest that appraisals and boredom are interwoven in a dynamically complex way. Though we used a modelling approach providing a detailed investigation of the appraisal‐boredom relationship, alternative model approaches and study designs can incorporate the function of time and timing more flexibly (see Asparouhov et al., 2018; Prasetio et al., 2020). A further limitation concerns the use of single‐item measures to assess comprehension, interest, and boredom. Whereas single‐item measures can be expected to allow for the assessment of students' appraisals and boredom in a way that minimizes interruption during learning (Bergkvist & Rossiter, 2007; Gardner et al., 1998; Wanous et al., 1997), we cannot entirely rule out the relevance of measurement error for the present findings. However, the applied modelling procedure, which constrained longitudinal relations across time lags, may help compensate for the potentially lower measurement precision at individual time points. Finally, the scope of the investigation was somewhat limited by its focus on boredom as the sole achievement emotion. On the other hand, one could argue that the complex and multidimensional nature of boredom makes it particularly well suited to the applied analytical approach.

In conclusion, our findings contribute to the growing body of research on the relationship between students' control‐value appraisals and achievement emotions, specifically the emergence of boredom during lessons. Notably, we employed a differentiated approach to disentangle the complex interplay between students' perceived interest, comprehension and boredom. From a teaching perspective, the study underscores the importance of students' momentary learning experiences. Regardless of individual dispositions, designing engaging and stimulating lessons appears to be one of the most effective strategies for preventing boredom. This conclusion primarily concerns within‐lesson fluctuations in boredom, interpreted relative to already controlled interindividual differences between students. At the same time, supporting students' comprehension emerges as a more delicate and complex task. The results suggest that while fostering general competencies to engage with learning content is important, it is equally essential to avoid underchallenging students in specific instructional moments. This challenge becomes even more critical in light of our finding that boredom, once it emerges, can enter into a reciprocal relationship with students' appraisals, potentially triggering a downwards spiral of disengagement and detachment from the learning process.

## AUTHOR CONTRIBUTIONS

**Richard Göllner:** Conceptualization; writing – original draft; methodology; writing – review and editing; formal analysis; visualization. **Kristina Kögler:** Conceptualization; writing – review and editing; funding acquisition; writing – original draft; investigation.

## FUNDING INFORMATION

The data used in this study resulted from a project funded by the University of Bamberg, Germany. The funds were dedicated to Kristina Kögler.

## CONFLICT OF INTEREST STATEMENT

The authors declare that they have no conflict of interest.

## ETHICS STATEMENT

No animals were involved. All procedures performed in studies involving human participants were in accordance with the ethical standards of the institutional and/or national research committee and with the 1964 Helsinki declaration and its later amendments or comparable ethical standards.

## INFORMED CONSENT

Informed consent was obtained from all individual participants included in the study.

## Data Availability

The dataset analysed during the current study is available from the corresponding authors upon request.

## Footnotes

^1^
The analysis of non‐linear relationships between comprehension and boredom was not part of the original set of hypotheses but was conducted as an exploratory analysis based on the suggestion of one of the reviewers.

^2^
Analysis codes of the prediction models are provided at https://osf.io/kyjwx/?view_only=2064526e4db94279a3bf1967173cb333 and data are available from both authors upon request.

^3^
The model including both comprehension and interest as predictors of boredom at the student and lesson levels demonstrated good fit to the data (*χ* ^2^(659) = 1003.93, *p* < .001; CFI = .97; TLI = .96; RMSEA = .027; SRMR_w_ = .044; SRMR_b_ = .060; scaling correction factor = .99).

## References

[bjep70048-bib-0001] Acee, T. W. , Kim, H. , Kim, H. J. , Kim, J.‐I. , Chu, H.‐N.‐R. , Kim, M. , Cho, Y. J. , & Wicker, F. W. (2010). Academic boredom in under‐ and over‐challenging situations. Contemporary Educational Psychology, 35(1), 17–27. 10.1016/j.cedpsych.2009.08.002

[bjep70048-bib-0002] Ahmed, W. , van der Werf, G. , Minnaert, A. , & Kuyper, H. (2010). Students' daily emotions in the classroom: Intra‐individual variability and appraisal correlates. British Journal of Educational Psychology, 80, 583–597. 10.1348/000709910X498544 20438661

[bjep70048-bib-0003] Arnold, M. B. (1960). Emotion and personality. Columbia University Press.

[bjep70048-bib-0004] Asparouhov, T. , Hamaker, E. L. , & Muthén, B. (2018). Dynamic structural equation models. Structural Equation Modeling, 25, 359–388. 10.1080/10705511.2017.1406803 29624092

[bjep70048-bib-0005] Bergkvist, L. , & Rossiter, J. R. (2007). The predictive validity of multiple‐item versus single‐item measures of the same constructs. Journal of Marketing Research, 44, 175–184. 10.1509/jmkr.44.2.175

[bjep70048-bib-0006] Berry, D. , & Willoughby, M. T. (2017). On the practical interpretability of cross‐lagged panel models: Rethinking a developmental workhorse. Child Development, 88, 1186–1206. 10.1111/cdev.12660 27878996

[bjep70048-bib-0007] Bieg, M. , Goetz, T. , & Hubbard, K. (2013). Can I master it and does it matter? An intraindividual analysis on control‐value antecedents of trait and state academic emotions. Learning and Individual Differences, 28, 102–108. 10.1016/j.lindif.2013.09.006

[bjep70048-bib-0008] Bong, M. , & Skaalvik, E. M. (2003). Academic self‐concept and self‐efficacy: How different are they really. Educational Psychology Review, 15, 1–40. 10.1023/A:1021302408382

[bjep70048-bib-0009] Camacho‐Morles, J. , Slemp, G. R. , Oades, L. G. , Pekrun, R. , & Morrish, L. (2019). Relative incidence and origins of achievement emotions in computer‐based collaborative problem‐solving: A control‐value approach. Computers in Human Behavior, 98, 41–49. 10.1016/j.chb.2019.03.035

[bjep70048-bib-0010] Camerman, E. , Kuppens, P. , Lavrijsen, J. , & Verschueren, K. (2024). Real‐time fluctuations in student emotions and relations with day of the week, time of the day, and teaching methods. Frontiers in Education, 9, 1470565. 10.3389/feduc.2024.1470565

[bjep70048-bib-0011] Csikszentmihalyi, M. (2000). Beyond boredom and anxiety. Jossey‐Bass.

[bjep70048-bib-0012] Daschmann, E. C. , Goetz, T. , & Stupnisky, R. H. (2011). Testing the predictors of boredom at school: Development and validation of the precursors to boredom scales. British Journal of Educational Psychology, 81, 421–440. 10.1348/000709910X526038 21770913

[bjep70048-bib-0013] Ellsworth, P. C. (2013). Appraisal theory: Old and new questions. Emotion Review, 5, 125–131. 10.1177/1754073912463617

[bjep70048-bib-0014] Ferla, J. , Valcke, M. , & Cai, Y. (2009). Academic self‐efficacy and academic self‐concept: Reconsidering structural relationships. Learning and Individual Differences, 19, 499–505. 10.1016/j.lindif.2009.05.004

[bjep70048-bib-0015] Forsblom, L. , Pekrun, R. , Loderer, K. , & Peixoto, F. (2022). Cognitive appraisals, achievement emotions, and students' math achievement: A longitudinal analysis. Journal of Educational Psychology, 114, 346–367. 10.1037/edu0000671

[bjep70048-bib-0016] Frenzel, A. C. , Pekrun, R. , & Goetz, T. (2007). Perceived learning environment and students' emotional experiences: A multilevel analysis of mathematics classrooms. Learning and Instruction, 17, 478–493. 10.1016/j.learninstruc.2007.09.001

[bjep70048-bib-0017] Gardner, D. G. , Cummings, L. L. , Dunham, R. B. , & Pierce, J. L. (1998). Single‐item versus multiple‐item measurement scales: An empirical comparison. Educational and Psychological Measurement, 58, 898–915. 10.1177/0013164498058006003

[bjep70048-bib-0018] Goetz, T. , Frenzel, C. A. , Pekrun, R. , Hall, N. C. , & Lüdtke, O. (2007). Between‐ and within‐domain relations of students' academic emotions. Journal of Educational Psychology, 99, 715–733. 10.1037/0022-0663.99.4.715

[bjep70048-bib-0019] Goetz, T. , Keller, M. M. , Lüdtke, O. , Nett, U. E. , & Lipnevich, A. A. (2020). The dynamics of real‐time classroom emotions: Appraisals mediate the relation between students' perceptions of teaching and their emotions. Journal of Educational Psychology, 112, 1243–1260. 10.1037/edu0000415

[bjep70048-bib-0020] Goetz, T. , Pekrun, R. , Hall, N. C. , & Haag, L. (2006). Academic emotions from a social‐cognitive perspective: Antecedents and domain specificity of students' affect in the context of Latin instruction. British Journal of Educational Psychology, 76, 289–308. 10.1348/000709905X42860 16719965

[bjep70048-bib-0021] Golle, J. , Flaig, M. , Jaggy, A.‐K. , & Göllner, R. (2022). Who's bored in school? Zeitschrift für Erziehungswissenschaft, 25, 1125–1149. 10.1007/s11618-022-01132-w

[bjep70048-bib-0022] Hamaker, E. L. , Kuiper, R. M. , & Grasman, R. P. P. P. (2015). A critique of the crosslagged panel model. Psychological Methods, 20(1), 102–116. 10.1037/a0038889 25822208

[bjep70048-bib-0023] Harackiewicz, J. M. , & Hulleman, C. S. (2010). The importance of interest: The role of achievement goals and task values in promoting the development of interest. Social and Personality Psychology Compass, 4, 42–52. 10.1111/j.1751-9004.2009.00207.x

[bjep70048-bib-0024] Hu, L. , & Bentler, P. M. (1999). Cutoff criteria for fit indexes in covariance structure analysis: Conventional criteria versus new alternatives. Structural Equation Modeling, 6, 1–55. 10.1080/10705519909540118

[bjep70048-bib-0025] Jackman, M. G. A. , Leite, W. L. , & Cochrane, D. J. (2011). Estimating latent variable interactions with the unconstrained approach: A comparison of methods to form product indicators for large, unequal numbers of items. Structural Equation Modeling, 18, 274–288. 10.1080/10705511.2011.557342

[bjep70048-bib-0026] Jia, F. , & Wu, W. (2023). A comparison of multiple imputation strategies to deal with missing nonnormal data in structural equation modeling. Behavior Research Methods, 55, 3100–3119. 10.3758/s13428-022-01936-y 36038813

[bjep70048-bib-0027] Kärner, T. , & Kögler, K. (2016). Emotional states during learning situations and students' self‐regulation: Process‐oriented analysis of person‐situation interactions in the vocational classroom. Empirical Research in Vocational Education and Training, 8, 12. 10.1186/s40461-016-0038-8

[bjep70048-bib-0028] Kögler, K. (2015). Langeweile in kaufmännischen Unterrichtsprozessen. Entstehung und Wirkung emotionalen Erlebens ungenutzter Zeitpotentiale. Peter Lang.

[bjep70048-bib-0029] Kögler, K. , & Göllner, R. (2018). Control‐value appraisals predicting students' boredom in accounting classes: A continuous‐state‐sampling approach. Empirical Research in Vocational Education and Training, 10(1), 4. 10.1186/s40461-018-0065-8

[bjep70048-bib-0030] Krannich, M. , Goetz, T. , Lipnevich, A. A. , Bieg, M. , Roos, A.‐L. , Becker, E. S. , & Morger, V. (2019). Being over‐ or underchallenged in class: Effects on students' career aspirations via academic self‐concept and boredom. Learning and Individual Differences, 69, 206–218. 10.1016/j.lindif.2018.10.004

[bjep70048-bib-0031] Krapp, A. (2002). Structural and dynamic aspects of interest development: Theoretical considerations from an ontogenetic perspective. Learning and Instruction, 12, 383–409. 10.1016/S0959-4752(01)00011-1

[bjep70048-bib-0032] Lazarides, R. , & Raufelder, D. (2021). Control‐value theory in the context of teaching: Does teaching quality moderate relations between academic self‐concept and achievement emotions? The British Journal of Educational Psychology, 91, 127–147. 10.1111/bjep.12352 32369196

[bjep70048-bib-0033] Loukomies, A. , Juuti, K. , & Lavonen, J. (2015). Investigating situational interest in primary science lessons. International Journal of Science Education, 37, 3015–3037. 10.1080/09500693.2015.1119909

[bjep70048-bib-0034] Marsh, H. W. , Lüdtke, O. , Robitzsch, A. , Trautwein, U. , Asparouhov, T. , Muthén, B. , & Nagengast, B. (2009). Doubly‐latent models of school contextual effects: Integrating multilevel and structural equation approaches to control measurement and sampling error. Multivariate Behavioral Research, 44, 764–802. 10.1080/00273170903333665 26801796

[bjep70048-bib-0035] Moors, A. (2013). On the causal role of appraisal in emotion. Emotion Review, 5, 132–140. 10.1177/1754073912463601

[bjep70048-bib-0036] Moors, A. (2017). The integrated theory of emotional behavior follows a radically goal‐directed approach. Psychological Inquiry, 28, 68–75. 10.1080/1047840X.2017.1275207

[bjep70048-bib-0037] Muthén, L. K. , & Muthén, B. O. (1998–2017). Mplus user's guide (8th ed.). Muthén & Muthén.

[bjep70048-bib-0038] Nett, U. E. , Bieg, M. , & Keller, M. M. (2017). How much trait variance is captured by measures of academic state emotions? A latent state‐trait analysis. European Journal of Psychological Assessment, 33, 239–255. 10.1027/1015-5759/a000416

[bjep70048-bib-0039] Nett, U. E. , Goetz, T. , & Hall, N. C. (2011). Coping with boredom in school: An experience sampling perspective. Contemporary Educational Psychology, 36, 49–59. 10.1016/j.cedpsych.2010.10.003

[bjep70048-bib-0040] Pekrun, R. (2006). The control‐value theory of achievement emotions: Assumptions, corollaries, and implications for educational research and practice. Educational Psychology Review, 18, 315–341. 10.1007/s10648-006-9029-9

[bjep70048-bib-0041] Pekrun, R. (2017). Emotion and achievement during adolescence. Child Development Perspectives, 11, 215–221. 10.1111/cdep.12237

[bjep70048-bib-0042] Pekrun, R. , Goetz, T. , Daniels, L. M. , Stupnisky, R. H. , & Perry, R. P. (2010). Boredom in achievement settings: Exploring control‐value antecedents and performance outcomes of a neglected emotion. Journal of Educational Psychology, 102, 531–549. 10.1037/a0019243

[bjep70048-bib-0043] Pekrun, R. , Hall, N. C. , Goetz, T. , & Perry, R. P. (2014). Boredom and academic achievement: Testing a model of reciprocal causation. Journal of Educational Psychology, 106, 696–710. 10.1037/A0036006

[bjep70048-bib-0044] Pekrun, R. , Lichtenfeld, S. , Marsh, H. W. , Murayama, K. , & Goetz, T. (2017). Achievement emotions and academic performance. Longitudinal models of reciprocal effects. Child Development, 88, 1653–1670. 10.1111/cdev.12704 28176309

[bjep70048-bib-0045] Pekrun, R. , & Loderer, K. (2020). Emotions and learning from multiple representations and perspectives. In P. Van Meter , A. List , D. Lombardi , & P. Kendeou (Eds.), Handbook of learning from multiple representations and perspectives (pp. 373–400). Taylor & Francis Group.

[bjep70048-bib-0046] Prasetio, B. H. , Tamura, H. , & Tanno, K. (2020). Deep time‐delay Markov network for prediction and modeling the stress and emotions state transition. Scientific Reports, 10, 18071. 10.1038/s41598-020-75155-w 33093631 PMC7581816

[bjep70048-bib-0047] Putwain, D. W. , Pekrun, R. , Nicholson, L. J. , Symes, W. , Becker, S. , & Marsh, H. W. (2018). Control‐value appraisals, enjoyment, and boredom in mathematics: A longitudinal latent interaction analysis. American Educational Research Journal, 55, 1339–1368. 10.3102/0002831218786689

[bjep70048-bib-0048] Putwain, D. W. , & Wood, P. (2023). Anxiety in the mathematics classroom: Reciprocal relations with control and value, and relations with subsequent achievement. ZDM Mathematics Education, 55, 285–298. 10.1007/s11858-022-01390-2

[bjep70048-bib-0049] Putwain, D. W. , Wood, P. , & Pekrun, R. (2022). Achievement emotions and academic achievement: Reciprocal relations and the moderating influence of academic buoyancy. Journal of Educational Psychology, 114, 108–126. 10.1037/edu0000637

[bjep70048-bib-0050] Raccanello, D. , Florit, E. , Brondino, M. , Rodà, A. , & Mason, L. (2022). Control and value appraisals and online multiple‐text comprehension in primary school: The mediating role of boredom and the moderating role of word‐reading fluency. British Journal of Educational Psychology, 92, 258–279. 10.1111/bjep.12448 34309018 PMC9292041

[bjep70048-bib-0051] Roseman, I. J. , & Smith, C. A. (2001). Appraisal theory: Overview, assumptions, varieties, controversies. In K. R. Scherer , A. Schorr , & T. Johnstone (Eds.), Appraisal processes in emotion: Theory, methods, research (pp. 3–19). Oxford University Press.

[bjep70048-bib-0052] Sembill, D. , Seifried, J. , & Dreyer, K. (2008). PDAs als Erhebungsinstrument in der beruflichen Lernforschung – Ein neues Wundermittel oder bewährter standard? [PDAs as assessment tools in research on vocational education and training – A new panacea or proven standard?] Empirische Pädagogik, 22, 64–77.

[bjep70048-bib-0053] Shao, K. , Pekrun, R. , Marsh, H. W. , & Loderer, K. (2020). Control‐value appraisals, achievement emotions, and foreign language performance: A latent interaction analysis. Learning and Instruction, 69, 101356. 10.1016/j.learninstruc.2020.101356

[bjep70048-bib-0054] Siegfried, C. , & Wuttke, E. (2019). Are multiple‐choice items unfair? And if so, for whom? Citizenship, Social and Economics Education, 18, 198–217. 10.1177/2047173419892525

[bjep70048-bib-0055] Silvia, P. J. (2005). What is interesting? Exploring the appraisal structure of interest. Emotion, 5, 89–102. 10.1037/1528-3542.5.1.89 15755222

[bjep70048-bib-0056] Tanaka, A. , & Murayama, K. (2014). Within‐person analyses of situational interest and boredom: Interactions between task‐specific perceptions and achievement goals. Journal of Educational Psychology, 106, 1122–1134. 10.1037/a0036659

[bjep70048-bib-0057] Tze, V. M. C. , Daniels, L. M. , & Klassen, R. M. (2016). Evaluating the relationship between boredom and academic outcomes: A meta‐analysis. Educational Psychology Review, 28, 119–144. 10.1007/s10648-015-9301-y

[bjep70048-bib-0058] van Tilburg, W. A. P. , & Igou, E. R. (2012). On boredom: Lack of challenge and meaning as distinct boredom experiences. Motivation and Emotion, 36, 181–194. 10.1007/s11031-011-9234-9

[bjep70048-bib-0059] Vodanovich, S. J. (2003). Psychometric measures of boredom: A review of the literature. Journal of Psychology, 137, 569–595. 10.1080/00223980309600636 14992349

[bjep70048-bib-0060] Wanous, J. P. , Reichers, A. E. , & Hudy, M. J. (1997). Overall job satisfaction: How good are single‐item measures? Journal of Applied Psychology, 82, 247–252. 10.1037/0021-9010.82.2.247 9109282

[bjep70048-bib-0061] Westgate, E. C. , & Wilson, T. D. (2018). Boring thoughts and bored minds: The MAC model of boredom and cognitive engagement. Psychological Review, 125, 689–713. 10.1037/rev0000097.supp 29963873

[bjep70048-bib-0062] Wild, K.‐P. , & Winteler, A. (1990). Fragebogen zum Interesse an wirtschaftlichen Zusammenhängen und an Computern [Questionnaire on interest in economic interrelationships and in computers]. Fakultät für Sozialwissenschaften der Universität der Bundeswehr München.

[bjep70048-bib-0063] Willett, J. B. , & Sayer, A. G. (1994). Using covariance structure analysis to detect correlates and predictors of individual change over time. Psychological Bulletin, 116, 363–381. 10.1037/0033-2909.116.2.363

